# Analytical Evaluation of Ground State Gradients in Quantum Electrodynamics Coupled Cluster Theory

**DOI:** 10.1021/acs.jctc.4c00763

**Published:** 2024-10-11

**Authors:** Marcus T. Lexander, Sara Angelico, Eirik F. Kjønstad, Henrik Koch

**Affiliations:** Department of Chemistry, Norwegian University of Science and Technology, 7491 Trondheim, Norway

## Abstract

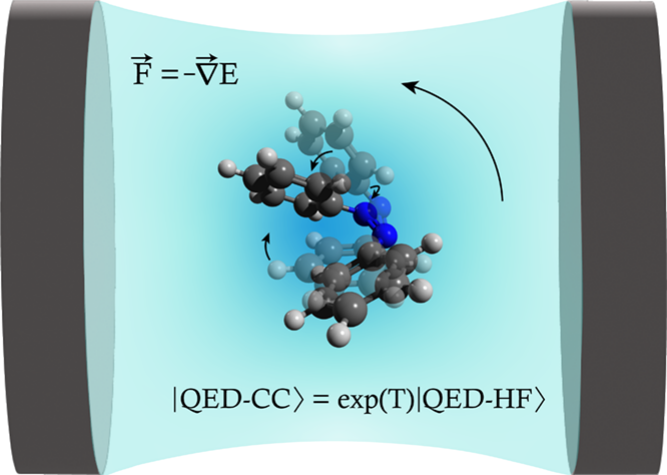

Analytical gradients of potential energy surfaces play a central role in quantum chemistry, allowing for molecular geometry optimizations and molecular dynamics simulations. In strong coupling conditions, potential energy surfaces can account for strong interactions between matter and the quantized electromagnetic field. In this paper, we derive expressions for the ground state analytical gradients in quantum electrodynamics coupled cluster theory. We also present a Cholesky-based implementation for the coupled cluster singles and doubles model. We report timings to show the performance of the implementation and present optimized geometries to highlight cavity-induced molecular orientation effects in strong coupling conditions.

## 1 Introduction

In the Born–Oppenheimer approximation, nuclei evolve on electronic potential energy surfaces, driven by the force given by the gradient of the electronic energy. The identification of relevant geometries on potential energy surfaces, as well as the study of chemical reactivity or orientational effects through molecular dynamics simulations, relies on the evaluation of molecular gradients of the potential energy surfaces.

An efficient evaluation of these gradients usually relies on an implementation of the analytical nuclear derivative of the electronic energy.^1,2^ Among several electronic structure methods, coupled cluster theory is well-known to provide a highly accurate description of dynamical correlation, both for ground and excited states, when the ground state is dominated by a single reference configuration.^3,4^ In addition, it is known to converge rapidly to the exact limit as one moves up the hierarchy of methods.^5,6^ Due to its increasingly feasible computational cost, its singles and doubles formulation (CCSD)^7^ is today extensively used for calculations of energies and different properties for medium sized systems. Many implementations of analytical gradients at the CCSD level have been reported over the past decades.^8−13^ More recently, decomposition methods for the electronic repulsion integrals have been used to further improve the efficiency of such gradient algorithms.^8,12−14^ In particular, the Cholesky decomposition method, which provides rigorous error thresholds, has recently become applicable to much larger systems due to algorithmic advances.^15−17^

In strong coupling conditions, the strong interactions between light and matter lead to the formation of hybrid light-matter states named polaritons. In these conditions, several experimental studies have shown modifications of e.g. ground state chemical and photochemical reactivity^18−22^ and supramolecular organization.^23−27^ A rationalization of such modifications, however, requires a detailed description of the quantum nature of both the molecule and the electromagnetic field. In this direction, many quantum chemistry *ab initio* methods have been generalized to quantum electrodynamics (QED). Recent examples include QED density functional theory,^28−31^ QED Hartree–Fock,^32,33^ QED configuration interaction,^32,34,35^ and QED coupled cluster theory.^32,36,37^ While the *ab initio* character of these methods provides a proper description of the molecular system, an accurate treatment of cavity-mediated reorientation effects, as well as changes in the equilibrium geometry, is needed in order to make more robust predictions.^38−40^ To this end, implementations of analytical gradients of some *ab initio* methods have already been implemented.^39,41,42^

In this work, we also move toward this end-goal, presenting a general formulation of analytical gradients for the ground state energy in QED coupled cluster (QED-CC) theory, along with an implementation at the QED-CC with single and double electronic excitations and single photonic excitations (QED-CCSD-1) level. We provide timings of the QED-CCSD-1 gradient evaluations in order to demonstrate the efficiency of the implementation, as well as optimized geometries in various systems to highlight the importance of cavity-induced orientation and relaxation effects.

## 2 Theory

### 2.1 QED Hamiltonian

In strong coupling conditions, the Hamiltonian must include the quantized electromagnetic field and its interactions with matter. Here, we describe such a system by means of the Pauli-Fierz Hamiltonian expressed within the length gauge representation of the dipole approximation.^38,43^ Moreover, we adopt the Born–Oppenheimer approximation, assuming that the wave function for the electronic and photonic degrees of freedom depends only on the positions of the nuclei. Working in the QED-HF coherent-state basis for a single mode of the electromagnetic field, we finally obtain the electronic-photonic Hamiltonian^32,44^
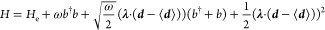
1where ***d*** is the dipole moment operator and ⟨***d***⟩ is its expectation value at the QED Hartree–Fock (QED-HF) level. The electromagnetic field is represented by a single harmonic oscillator with frequency ω and photon creation and annihilation operators denoted by *b*^†^ and *b*.

The first two terms of eq 1 describe the molecular electronic Hamiltonian, *H*_e_, and the energy of the quantized electromagnetic field, respectively. The third term in *H* describes the bilinear interaction between the molecular system and the displacement field. The final term is the dipole self-energy, which ensures that the Hamiltonian is bounded from below.^45,46^ The coupling strength of the field is denoted by , where *V* is the quantization volume and **ε** the polarization vector. To simplify the notation, we will let *d* = **λ** · ***d*** in the following.

By expanding the electronic Hamiltonian in the second quantization formalism, eq 1 can be rewritten as
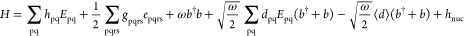
2Here *h*_nuc_ is the nuclear repulsion energy, and we have defined the dipole moment operator, including both an electronic part *d*_pq_^e^ and a nuclear part *d*_N_, as
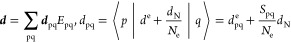
3*N*_e_ is the number of electrons of the molecule. The indices *p*, *q*, *r*, *s* denote molecular orbitals (MOs), and
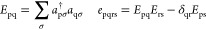
4where *a*^†^ and *a* are the creation and annihilation operators for the electrons, respectively. Moreover, the one- and two-electron integrals *h*_pq_ and *g*_pqrs_ are dressed electronic integrals that include contributions from the electromagnetic field. Denoting the electronic one- and two-electron integrals as *h*_pq_^e^ and *g*_pqrs_^e^, we have
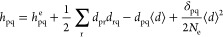
5
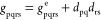
6Note that, when introducing the second quantization formalism for the electronic Hamiltonian, we have implicitly made use of the complete basis set approximation (i.e., we have approximated the square of the second quantization dipole moment operator *d̂*^2^ = *d̂* · *d̂*).^44^ The generalization of the current implementation explicitly including the quadrupole moment is straightforward. Moreover, since in our implementation, which builds on that in ref (32), we have included the nuclear dipole moment in the total dipole operator, we will need to account for terms originating from its derivative in the evaluation of the molecular gradient.

### 2.2 QED-CC

In QED-CC, coupled cluster theory is extended to include the interactions between the electrons and the quantized electromagnetic field. The wave function is obtained by applying the exponential of the cluster operator *T* to a reference wave function. This is typically chosen to be the QED-HF wave function, where the HF orbitals are calculated from the Hamiltonian in eq 1:^32^
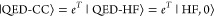
7The cluster operator is defined as
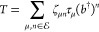
8where
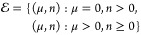
9denotes the set of excitation operators in *T*. In particular,  contains all elements in the projection manifold except |HF, 0⟩. Here, μ labels the electronic excitations, with μ = 0 denoting the HF state, and *n* denoting the photonic excitation. We can partition the cluster operator into purely electronic, purely photonic, and mixed excitation operators,
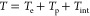
10with
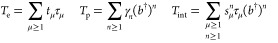
11Moreover, we will refer to ζ_μ*n*_ = {*t*_μ_, γ_*n*_, *s*_μ_^*n*^} as the QED-CC amplitudes. We can determine the QED-CC energy and amplitudes by projecting the Schrödinger equation on the excitation set^32^:
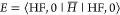
12
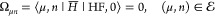
13where *H̅* = *e*^–*T*^*He^T^* is the similarity transformed Hamiltonian.

While these equations provide expressions for the energy and the amplitudes, the direct evaluation of the molecular gradient as total derivative of the energy of eq 12 is complicated and usually avoided. For this reason, in the next section, we describe the Lagrangian formalism, which is commonly used to derive analytical expressions for the molecular gradients in coupled cluster theory and other electronic structure methods.^47−50^

### 2.3 Lagrangian Formalism

In coupled cluster theory, the dependence of the ground state energy on the amplitudes is non-variational; that is, the energy is not stationary with respect to the amplitudes. As a consequence, we cannot invoke the usual Hellmann–Feynman theorem to calculate nuclear gradients. Nevertheless, we can avoid explicitly evaluating the derivatives of the wave function parameters by adopting the Lagrangian formalism (or Z-vector technique).^49,50^

In this formalism, we consider a function (for example, the energy *E*) that depends on some parameters **λ**, which should not be confused with the light-matter coupling vector **λ** described above. These parameters are determined by imposing a set of conditions {*e*_p_ = 0}. To each of these constraints *e*_p_, we can now associate a Lagrangian multiplier λ̅_p_ and define a Lagrangian  as
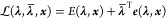
14where ***x*** denotes the nuclear coordinates. In order to keep the notation simple, we will not make the dependence on ***x*** explicit in the following.

To enforce the set of constraints ***e*** = 0 and determine the parameters **λ**, we require that the Lagrangian is stationary with respect to the multipliers **λ̅**. The multipliers, instead, are determined by requiring that  is stationary with respect to the parameters **λ**:
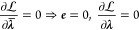
15When these equations are satisfied, the Lagrangian is by definition equal to the energy at every value of ***x***. As a consequence, the total derivative of the energy with respect to ***x*** can be evaluated as the partial derivative of the Lagrangian:
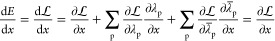
16In this way, the molecular gradient can be evaluated without needing to evaluate the derivative of the parameters with respect to the nuclear coordinates.

Applying this formalism to the QED-CC ground state energy, we can define the Lagrangian as
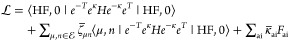
17Here, the first term is the QED-CC ground state energy, and the second term corresponds to the Ω_μ*n*_ equations and the associated multipliers ζ̅_μ*n*_. The last term enforces the QED-HF equations through the associated multipliers κ̅_*ai*_. Moreover, we have made the ***x***-dependence of the orbitals explicit by introducing the orbital rotation operator κ. By assumption, the QED-HF orbitals are optimized, and κ = 0, at the geometry where the gradient is evaluated.^50^ Finally, we can rewrite the QED-CC Lagrangian in a more compact form by introducing the dual ground state vector ⟨Λ|:
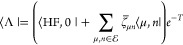
18
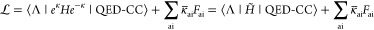
19where we have defined *H̃* = *e*^κ^*He*^–κ^.

### 2.4 Gradient Expression

Using eqs 16 and 19, the molecular gradient can be expressed as
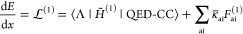
20where we have denoted nuclear derivatives with ^(1)^.

So far, we have taken care of the constraints for the QED-HF and QED-CC equations, which are imposed via the Lagrangian. However, when evaluating molecular gradients, we must also ensure that the MOs are kept orthonormal at all nuclear geometries, since we implicitly assume this in all our derivations. In fact, the Hamiltonian and other operators, as well as the state vectors, are represented in terms of creation and annihilation operators (*a*_pσ_^†^, *a*_pσ_) that are associated with a set of orthonormal MOs (ϕ_p_).^4^ To account for orthonormality, we employ an orbital connection. Given a reference geometry ***x***_0_, at which we will calculate the gradient, we can consider some unmodified MOs (UMOs) at a neighboring geometry ***x***,
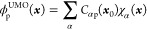
21formed by freezing the orbital coefficients *C*_αp_ at ***x***_0_. These UMOs are not orthonormal, and we denote the overlap matrix as *S*_pq_(***x***) = ⟨ϕ_p_^UMO^(***x***)|ϕ_q_^UMO^(***x***)⟩. An orbital connection ***T*** restores orthonormality by transforming the UMOs into a set of orthonormal MOs (OMOs),
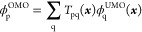
22In this paper, we adopt the symmetric connection ***T*** = ***S***^–1/2^.^51^

From eq 22, it follows that we can separate the derivatives of the Hamiltonian into two contributions. The first one arises from the UMOs and the second one from the ***x***-dependence of ***T***. To evaluate the latter, we note that ***T***^†^***ST*** = ***TST*** = **1**, and take the derivative at ***x***_0_ (where ***S*** = **1**). We then find
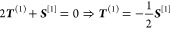
23The ^[1]^ notation denotes that the derivative is taken in the UMO basis. Finally, we can consider the one-electron part of the Hamiltonian, *h*. We can write the *h* derivative at ***x***_0_ as
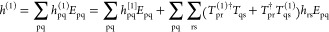
24Now, since *T*_pq_(***x***_0_) = δ_pq_, we can simplify this expression to
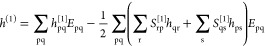
25Expressing the two one-index transformations by {···}, we find that
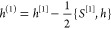
26In the case of the two-electron part of the Hamiltonian, *g*, we obtain four one-index transformations between *S*^[1]^ and *g* when taking the derivative. Collecting the one- and two-electron terms with the notation {···}, we can write the derivative of the Hamiltonian operator as
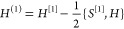
27Terms arising from the one-index transformations are referred to as “reorthonormalization terms” and will be considered in more detail below. In the above, we have used that the creation and annihilation operators can be considered independent of ***x*** in the case of energy derivatives.^51^

The final expression for the gradient reads
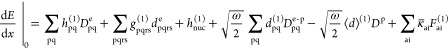
28where we have introduced the densities
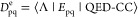
29
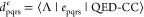
30
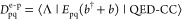
31
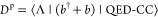
32The molecular gradient thus depends on electronic, photonic, and mixed electronic-photonic densities. Note that the densities depend on both the QED-CC amplitudes from the cluster operator and multipliers from the dual vector (see eq 19). These multipliers are obtained by solving a set of response equations described in the following section. Autogenerated programmable expressions for the densities are given in the Supporting Information.

### 2.5 Response Equations

In order to evaluate the nuclear gradient, we first need to determine the Lagrangian multipliers by solving two sets of response equations. First, by considering the derivative of the Lagrangian with respect to the QED-CC amplitudes, one gets the response (or stationarity) equation for the QED-CC multipliers **ζ̅**:
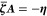
33Here, the Jacobian ***A*** and the **η** vectors are the analogs of the standard equation of motion coupled cluster quantities,^32^
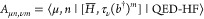
34
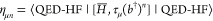
35The derivative of the Lagrangian with respect to the orbital rotation parameters, instead, gives the response equation for the **κ̅** multipliers:
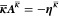
36Here, ***A***^**κ̅**^ is the QED-HF Hessian,
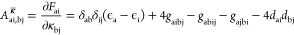
37while **η**^**κ̅**^ can be expressed as
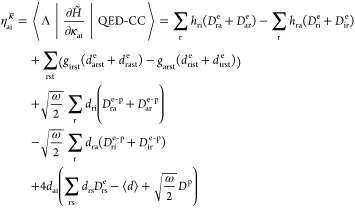
38Here, we have used
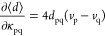
39with *v*_p_ = 1 if *p* denotes an occupied orbital and *v*_p_ = 0 otherwise. All partial derivatives are evaluated at **κ** = 0. Note that while in the first two lines of eq 38 the standard definition^11^ of the **η**^**κ̅**^ vector is obtained (albeit in terms of the dressed one- and two-electron integrals), new contributions due to the quantized electromagnetic field arise in the last two lines.

## 3 Implementation

In the following, we will work at the QED-CCSD-1 level of theory, where *T*_e_ includes single and double electronic excitations and *T*_p_ includes single photonic excitations. Additionally, *T*_int_ includes simultaneous electron-photon excitations obtained by combining the included electronic and photonic excitations:
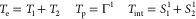
40where
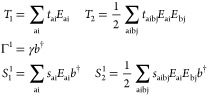
41Here, *i*, *j* denote occupied orbitals and *a*, *b* denote virtual orbitals. To determine the ground state energy and amplitudes, we solve eq 13 using the projection set
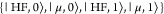
42where μ is restricted to single and double excitations.

Our implementation of QED-CCSD-1 gradients builds on the EOM-CCSD nuclear gradient implementation by Schnack-Petersen et al.,^13^ which makes use of Cholesky-decomposed two-electron integrals to evaluate the gradient. Here, we highlight the aspects of this implementation that are most relevant to the present work and refer to ref (13) for more details. The two-electron integral matrix is sparse and positive definite and admits to a Cholesky decomposition, which can be expressed directly or in a resolution-of-identity form,^52^
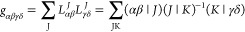
43where the *J* and *K* indices denote AO index pairs that are referred to as Cholesky pivots and α, β, γ, δ denote AO indices. Using the resolution of identity form, we can write the contribution of the two-electron terms in the energy (in the MO basis) as
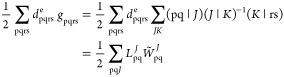
44where we have defined
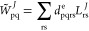
45The resolution-of-identity form is particularly useful because it allows us to evaluate nuclear derivatives without determining the derivatives of the Cholesky vectors (*L*_αβ_^*J*^).^13,14^ Moreover, the introduction of the intermediate *W̃*_pq_^*J*^ allows us to avoid storing the memory-intensive four-index block of the density matrix (e.g., *d*_abcd_), whose contributions are instead stored in the three-index tensor *W̃*_pq_^*J*^.^13^

## 3.1 Response Equations

In the case of the amplitude response, ***A*** and **η** have already been implemented, and we refer the reader to ref (32) for more details. In the case of the orbital relaxation, κ̅_ai_ is determined by solving eq 36. The implementation of the right-hand side makes use of the  intermediate and the permutation operator *P*_ai_ (*P*_ai_*X*_ai_ = *X*_ia_):
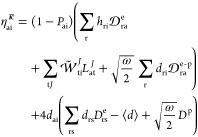
46where we have introduced the symmetrized quantities
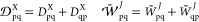
47Finally, the implementation of the QED-HF Hessian transformation, starting from an existing HF implementation,^13^ is straightforward provided that the one- and two-electron integrals are properly redefined to include the QED contributions.

## 3.2 Nuclear Gradient

Once the response equations have been solved, the nuclear gradient can be calculated. As mentioned before, this is usually split in two contributions. At first, UMOs contributions are considered. These include one- and two-electron contributions, both from the energy and the orbital relaxation terms, as well as contributions coming from the bilinear term of the Hamiltonian. The one-electron and bilinear contributions are straightforward and will not be discussed further. Below, we describe the two-electron and reorthonormalization terms in more detail, emphasizing the required modifications to obtain the QED-CCSD-1 quantities starting from an existing CCSD implementation.

### 3.2.1 Two-Electron Contributions

Using the resolution-of-identity form in eq 43, we find that the two-electron contributions to the gradient (in the UMO basis) can be written
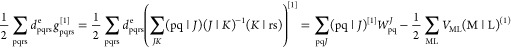
48where we have defined the Cholesky intermediates
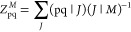
49
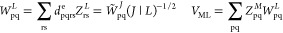
50A similar strategy can be used to evaluate the two-electron contributions to the orbital relaxation gradient. In this case, similar intermediates to *W*_pq_^*J*^ are defined from the contraction of *Z*_pq_^*J*^ with κ̅_ai_. A detailed description of these intermediates can be found in ref (13). Note that the *W*_pq_^*L*^ intermediates here introduced are calculated from *W̃*_pq_^*L*^. In this way, contractions with the two-electron density matrix only need to be evaluated once.

From the given list of the Cholesky pivots {*J*}, we can evaluate the gradient by requesting (αβ|*J*)^(1)^ and (*J*|*K*)^(1)^ from an integral program, then transforming the α and β indices to the MO basis, and, finally, evaluating the contractions in eq 48 using the precalculated Cholesky intermediates. Hence, the memory-intensive four-index blocks of the density matrix do not need to be stored, as smaller batches of these densities can be constructed and immediately contracted with the Cholesky vectors, yielding the much less memory-intensive three-index Cholesky intermediates.^13^

These advantages generalize straightforwardly to QED-CCSD. Here, the derivative of the dressed two-electron integral matrix *g*_pqrs_ can be expanded as
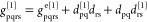
51In this expression, the derivative of the dipole moment contains both electronic and nuclear contributions, and we can further expand this term as
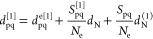
52In the evaluation of the gradient, the derivatives (αβ|*J*)^(1)^ and (*J*|*K*)^(1)^ are calculated in terms of AO-shells. In particular, a given *J* represents a pair of AOs γδ and the integral program calculates the integrals as (*AB*|*CD*), where α is in the shell A, β is in the shell B, and so on. Now, for the undressed integral *g*_*ABCD*_^e^ = (*AB*|*CD*)^e^, the derivative is non-zero only when differentiating with respect to one of the atoms in the shell quartet. As a consequence, its derivative only has 12 non-zero components. This reduced dimensionality is exploited to reduce computational costs.^13^ However, the generalization of the algorithm to the QED case requires the introduction of the derivative of the dipole moment in eq 52. Here, we note that the first two terms have 6 non-zero components, while the third one, involving the derivative of the nuclear dipole moment, has 3N nonzero components, where N is the number of atoms in the molecule. As a consequence, the generalization to the QED case requires a redefinition of (αβ|*J*)^(1)^ and (*K*|*L*)^(1)^ to include the QED contributions with 6 non-zero components and a subsequent separate treatment of the *d*_N_^(1)^ contributions. This will also be the case for the two-electron part of the orbital relaxation gradient.

From eqs 51 and 52, the *d*_N_^(1)^ contributions to the two-electron gradient are
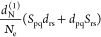
53In order to treat the *d*_N_^(1)^ contributions, we could insert this expression into eq 48. However, this is equivalent to explicitly considering these terms without introducing the Cholesky decomposition. Here, we present only the final expression of the *d*_N_ contributions to the two-electron gradient, while we show this equivalence in the Supporting Information.

From eq 53, the additional terms needed for the two-electron gradient are
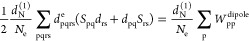
54where we have defined
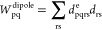
55Note that, by using
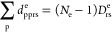
56we can simplify eq 54 and rewrite it as
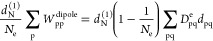
57Finally, using eq 53, the nuclear contributions to the orbital relaxation terms read
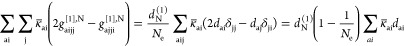
58

### 3.2.2 Reorthonormalization

Above, we have focused on the UMO contributions to the molecular gradient. The last terms to account for are the reorthonomalization contributions, which arise from the derivative of the orbital connection. These contributions are usually expressed in the MO basis in terms of a generalized Fock matrix  such that the gradient contribution takes the form . In the QED-CCSD-1 case,  can be defined as
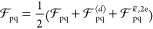
59where
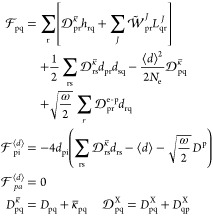
60Here, we have separated the two-electron contributions to the orbital relaxation in . Since the *d*_N_^(1)^ part of the derivative dipole moment has no reorthornormalization contributions,  is the same as the one in the CCSD case, with dressed two-electron integrals. We refer to ref (13) for this term. The other two terms are given in the Supporting Information.

**Figure 1:**
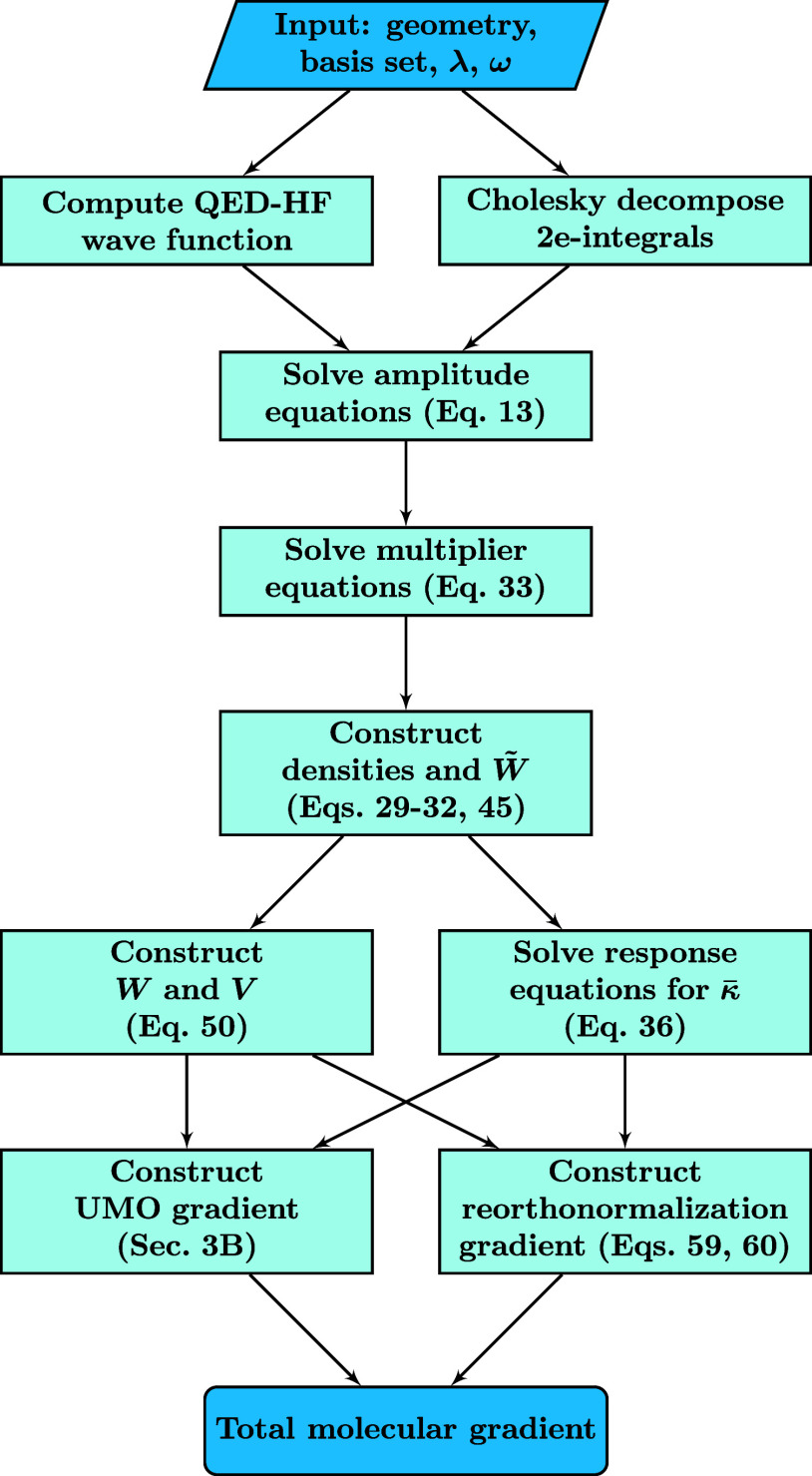
Procedural summary for the evaluation of the analytical gradient.

This concludes the presentation of the different terms involved in the evaluation of the molecular gradient. To give an overview of the necessary steps, a procedural summary for the evaluation of the gradient is presented in Figure 1.

## 4 Results and Discussion

To illustrate the efficiency and possible applications of the QED-CCSD-1 gradients, we present timings of the implementation as well as optimized geometries for a few molecular systems. The QED-CCSD-1 gradient has been implemented in a development branch of the eT 2.0 program.^53^ Geometry optimizations were performed using an interface to the geomeTRIC package,^54^ which allows for efficient optimization of molecular geometries, as well as global orientation, which is crucial when optimizing molecular systems in the presence of an external field.^39^ For the QED-CCSD-1 calculations, the cavity frequency is set equal to the first bright excitation energy, calculated at the CCSD level. The coupling strength is set to ||**λ**|| = 0.05 au. All geometry optimizations were performed using the cc-pVDZ basis set. The coordinates for the optimized geometries are provided in a separate repository.^55^ Timings were run on an Intel(R) Xeon(R) Platinum 8380 system with 80 cores and 2 TB of memory. Finally, a comparison of a numerical (4-point finite difference stencil) and analytical evaluation of the gradients on H_2_O—He is provided in the Supporting Information.

## 4.1 Timings

**Figure 2:**
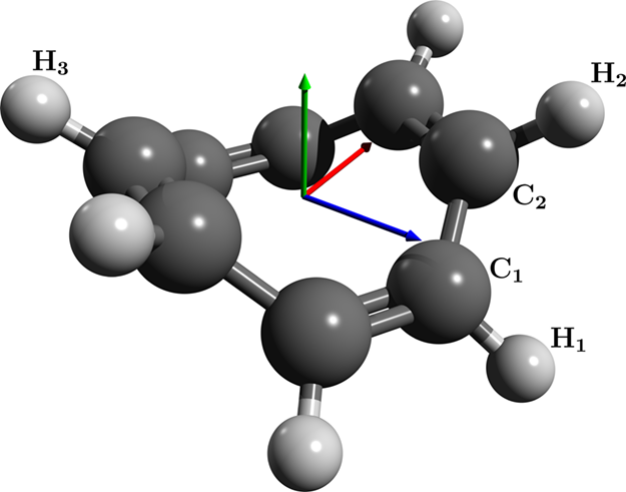
Optimized geometry for the cyclooctatetraene molecule using QED-CCSD-1 with λ = 0.05 au and the cc-pVDZ basis set. The polarization vector is indicated by the green arrow.

**Figure 3:**
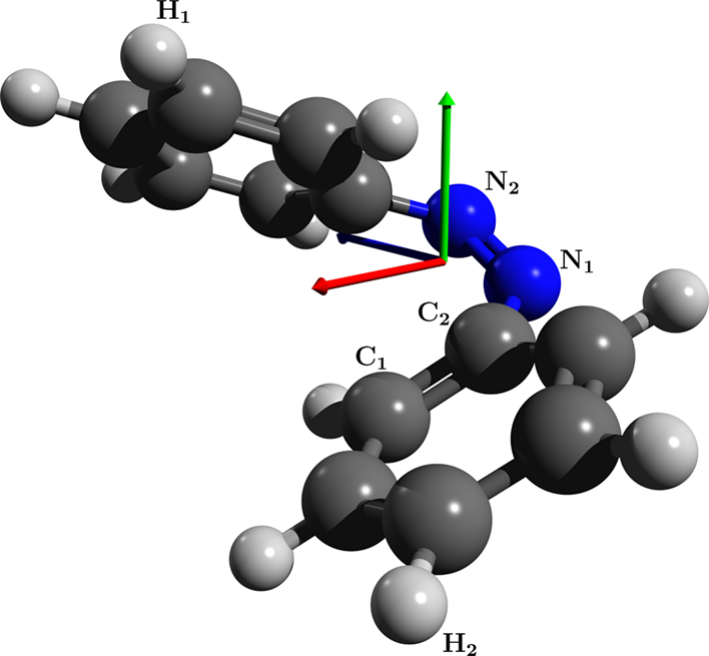
Optimized geometry for the cis-isomer of azobenzene using QED-CCSD-1 with λ = 0.05 au and the cc-pVDZ basis set. The polarization vector is indicated by the green arrow.

**Figure 4:**
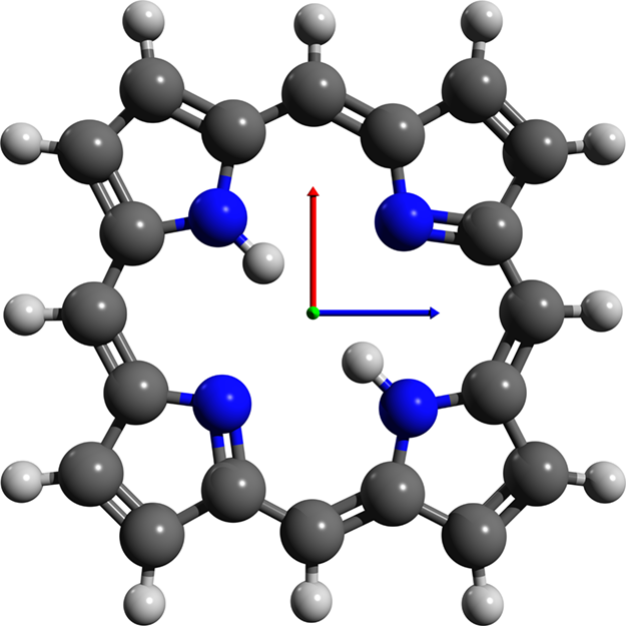
Optimized geometry for the porphine molecule using QED-CCSD-1 with λ = 0.05 au and the cc-pVDZ basis set. The molecule is planar, with the polarization vector perpendicular to the plane of the molecule, indicated by the green arrow pointing out of the figure.

We compare timings for the evaluation of the gradient with CCSD and QED-CCSD-1 for three molecular systems. We note that both the CCSD and QED-CCSD-1 calculations were run with the same thresholds for Cholesky decomposition of the two-electron integrals, as well as for the convergence of the amplitude and multiplier equations. As test systems, we consider cyclooctatetraene, with the aug-cc-pVTZ basis set, and azobenzene and the porphine molecule with the cc-pVDZ basis sets. The selected molecules are shown in Figures 2, 3, and 4. Timing data are presented in Table 1. The time to solve the multiplier equations (eq 33) is not included in the gradient time as this is not strictly part of the gradient evaluation, but it represents a significant part of the total time of the calculation.

**Table 1:** Timings for a Single Gradient Evaluation Using CCSD and QED-CCSD-1

	*n*_occ_/*n*_vir_	method	*t*_2e-density_	*t*_gradient_	*t*_total_	*t*_gradient_/*t*_total_
cyclooctatetraene	28/524	CCSD	243 s	324 s	3154 s	(10.3%)
(aug-cc-pVTZ)		QED-CCSD-1	522 s	611 s	7959 s	(7.9%)
azobenzene	48/198	CCSD	9.7 s	22.9 s	267.1 s	(8.6%)
(cc-pVDZ)		QED-CCSD-1	25.7 s	41.9 s	872.1 s	(4.8%)
porphine	81/325	CCSD	140 s	267 s	3303 s	(8.1%)
(cc-pVDZ)		QED-CCSD-1	406 s	530 s	13821 s	(3.8%)

As expected, we see that in all cases, the time for the molecular gradient evaluation is longer for QED-CCSD-1 than for CCSD. However, the evaluation of the gradient always constitutes a very small fraction of the total time. For example, in cyclooctatetraene, the evaluation of the analytical molecular gradient only represents 8% of the time of the whole calculation. In the evaluation of the QED-CCSD-1 gradient, the additional cost compared to CCSD is found to be almost exclusively due to the QED terms in the two-electron density matrix. The remaining QED contributions add a negligible cost compared to CCSD.

For medium-sized systems with relatively small basis sets, a significant part of the calculation time is given by terms (with scaling *o*^3^*v*^3^ or lower) that do not contribute to the asymptotic *o*^2^*v*^4^ scaling of the method. This can be seen when the number of virtual orbitals is relatively small, as for azobenzene and porphine, where the QED-CCSD-1 calculation takes about four times longer than the CCSD calculation. For cyclooctatetraene with aug-cc-pVTZ, on the other hand, the cost of QED-CCSD-1 is approximately twice the cost of CCSD, as expected from the asymptotic scaling of the two methods. Further optimizations of the iterative terms in QED-CCSD-1 may lower the relative cost for medium-sized systems.

## 4.2 Geometry Optimization

### 4.2.1 Cyclooctatetraene

The anti-aromatic molecule cyclooctatetraene has a boat shape in its ground state geometry. Starting from the optimized CCSD geometry in a random orientation, we find that the field causes the molecule to reorient such that the plane of the boat lies perpendicular to the cavity polarization. This allows the molecule to minimize its spatial extent along the polarization axis, which lowers the total energy. The optimized geometry is shown in Figure 2. As expected, in addition to the reorientation, we see a slight flattening of the molecule along the polarization axis. In particular, we find a slight increase in the distance between two opposite hydrogen atoms (H_2_ and H_3_) and a slight decrease in the dihedral angle γ between two adjacent CH groups (H_1_, C_1_, C_2_, H_2_). Values of these selected bond lengths and angles are given in Table 2.

**Table 2:** Selection of Optimized Internal Coordinates for Cyclooctatetraene^a^

method	r_H_2_–H_3__ (Å)	γ (°)
CCSD	5.22	48.4
QED-CCSD-1	5.25	47.1

a γ is the dihedral angle between H_1_–C_1_–C_2_–H_2_.

### 4.2.2 Azobenzene

As a second example, we consider the cis-isomer of azobenzene. As in the previous case, starting from the optimized CCSD geometry in a random orientation, we find that the molecule rotates in order to minimize its energy. As can be seen in Figure 3, however, the optimized molecular structure preserves a certain angle relative to the polarization direction. In Table 3, we report this relative orientation of the molecule as the angle θ between the N–N bond and the direction of the cavity field. When introducing the cavity field, we also observe a rotation of the phenyl groups around the C–N bonds, which leads the groups to be more perpendicular to the polarization. In this case, we report in Table 3 both the angle between the plane of the phenyl group and the polarization direction ϕ and the dihedral angle α between C_1_, C_2_, N_1_ and N_2_ (see Figure 3).

**Table 3:** Selection of Optimized Internal Coordinates for *cis*-Azobenzene^a^

method	r_H_1_–H_2__ (Å)	α (°)	θ (°)	ϕ (°)
CCSD	5.60	57.1		
QED-CCSD-1	5.19	52.0	61.3	30.7

a α is the C_1_–C_2_–N_1_–N_2_ dihedral angle, θ is the angle between the N–N bond and the polarization vector, and ϕ is the angle between the C_1_–C_2_ phenyl group and the polarization direction.

### 4.2.3 Porphine

Porphine is the base-structure for large categories of biological molecules called porphyrins and chlorins. The optimized geometry is shown in Figure 4. Again, we find that the molecule orients itself to become perpendicular to the polarization vector. In contrast to the other two systems, however, the internal geometry of this molecule does not change noticeably when coupling to the field. A different behavior, however, might be obtained considering more than one cavity mode with different polarization directions as the plane of the molecule can not be perpendicular to both polarization directions. In this case, the components of the polarization that lie in the plane of the molecule might cause more visible internal geometry changes.

## 5 Conclusions

In this paper, we presented a formulation of ground state analytical gradients for QED-CC together with an efficient Cholesky-based implementation at the QED-CCSD-1 level. Using the resolution-of-identity form, we avoid the evaluation of the derivative of the Cholesky vectors.^13,14^ Moreover, building on an existing implementation of CCSD gradients,^13^ we reduce the memory usage by using an on-the-fly construction of the intermediates involving the *v*^4^ block of the density matrix. Timings for a single gradient evaluation show that the calculation of the analytical gradient requires less than 10% of the total time of the QED-CCSD-1 calculation.

Moreover, we optimized the geometries of cyclooctatetraene, azobenzene, and porphine in an optical cavity. In all cases, we allowed for rotations of the molecules, thus showing the reorientation of the system with respect to the polarization of the field. This highlights the importance of including cavity-induced effects when determining optimal geometries, as already suggested by some recent studies.^38−40^

Given the well-established accuracy of coupled cluster theory and the efficiency of the implementation, we believe that the implementation will prove to be a useful tool for determining equilibrium geometries in optical cavities. Moreover, the implementation can also be used to parametrize classical force fields or to perform ground state *ab initio* molecular dynamics simulations of cavity-induced orientational effects in molecular ensembles.

## Supporting Information Available

Comparison with numerical gradients, notes on the treatment of the derivative of the nuclear dipole moments, derivations of the reorthonormalization terms and programmable expressions for the densities (PDF)
