# Supplementary material for: Analytical Evaluation of Ground State Gradients in Quantum Electrodynamics Coupled Cluster Theory
Source: J Chem Theory Comput. 2024 Oct 11;20(20):8876–85. doi: 10.1021/acs.jctc.4c00763 (PMC11500291; doi:10.1021/acs.jctc.4c00763)
Supplement: Supplementary file 1 — ct4c00763_si_001.pdf [file ct4c00763_si_001.pdf]

Supporting information for "Analytical evaluation of ground state gradients in quantum electrodynamics coupled cluster theory"

Marcus T. Lexander,[1,2] Sara Angelico,[1,2] Eirik F. Kjønstad,[1] and Henrik Koch[1,a)]

[1)] *Department of Chemistry, Norwegian University of Science and Technology, 7491 Trondheim*

[2)] *These authors contributed equally to this work*

(Dated: 14 August 2024)

[a)] henrik.koch@ntnu.no

CONTENTS

S1. Comparison of the analytical and numerical gradients S3

S2. Notes on the treatment of the nuclear contributions to the Cholesky vectors S4

S3. Derivations for the reorthonormalization contributions S6

A. Dressed one-electron integral S7

B. Reorthonormalization of the bilinear term S8

C. Orbital relaxation (Fock matrix) S9

S4. Density matrix expressions S10

A. One-electron density S10

B. One-electron-one-photon density S10

C. One-photon density S11

D. Two-electron density S11

References S15

S1. COMPARISON OF THE ANALYTICAL AND NUMERICAL GRADIENTS

To test the correctness of the implementation of the analytical gradient, we compare the analytical and the numerical gradients for H_2O-He. The geometry of the system can be found in Table S1. The gradient was calculated with the cc-pVDZ basis set. The frequency, coupling strength and polarization directions are $\omega = 0.5$ a.u., $\lambda = 0.05$ a.u., $\boldsymbol{\epsilon} = [0.577350, 0.577350, 0.577350]$. The numerical gradient is calculated with a five point stencil, with $h = 1 \cdot 10^{-4}$ Å. The maximum deviation between the analytical and numerical gradient is $4 \cdot 10^{-9}$.

TABLE S1. Comparison of numerical and analytical gradients. The gradients are calculated using the cc-pVDZ basis set. Numerical gradients were evaluated with a five-point stencil using a displacement of $h = 1 \cdot 10^{-4}$ Å. The molecular geometry is given in Å, while the gradients are expressed in atomic units. The a.u. to Å conversion factor used is 0.52917721092.

		Molecular geometry (Å)	
H	0.866 810 000	0.601 440 000	5.000 000 000
H	−0.866 810 000	0.601 440 000	5.000 000 000
O	0.000 000 000	−0.075 790 000	5.000 000 000
He	0.100 000 000	−0.020 000 000	7.530 000 000
		Analytical gradient (a.u.)	
H	0.074 446 196	0.047 170 451	0.000 360 463
H	−0.074 310 167	0.046 932 779	0.000 053 345
O	−0.000 031 410	−0.094 104 796	0.002 633 639
He	−0.000 104 618	0.000 001 566	−0.003 047 447
		Numerical gradient (a.u.)	
H	0.074 446 196	0.047 170 450	0.000 360 459
H	−0.074 310 166	0.046 932 781	0.000 053 346
O	−0.000 031 410	−0.094 104 796	0.002 633 640
He	−0.000 104 614	0.000 001 565	−0.003 047 451

S2. NOTES ON THE TREATMENT OF THE NUCLEAR CONTRIBUTIONS TO THE CHOLESKY VECTORS

In the following, we will make use of the following identities:

$$\sum_{JK} S_J (J|K)^{-1}(K|pq) = S_{pq} = \delta_{pq} \qquad \sum_{JK} d_J (J|K)^{-1}(K|pq) = d_{pq} \tag{1}$$

We will now prove the first identity. A similar derivation can be followed to obtained the second one.

To prove the first identity, we start by considering $W_K = \sum_J S_J (J|K)^{-1}$. Inverting this relation, we get

$$\sum_K W_K (K|L) = S_L. \tag{2}$$

Using that L corresponds to a pair of AOs $\gamma\delta$ and transforming them to the MO basis we obtain

$$\sum_K \sum_{\gamma\delta} W_K S_{K,\gamma\delta} C_{\gamma p} C_{\delta q} = S_{pq} \tag{3}$$

$$\sum_K W_K S_{K,pq} = \delta_{pq}. \tag{4}$$

Substituting the definition of W_K we find

$$\sum_{KJ} S_J (J|K)^{-1}(K|pq) = \delta_{pq} \tag{5}$$

We can now consider the $d_N^{(1)}$ contributions to the two-electron gradient. As shown in eq. (48), these can be expressed as

$$\begin{aligned}\frac{1}{2}\sum_{pqrs} d^e_{pqrs} g^{[1]}_{pqrs} &= \sum_{pqJ} (pq|J)^{[1]} \Bigg(\sum_{rsK} d_{pqrs} (J|K)^{-1}(K|rs) \Bigg) \\ &\quad - \frac{1}{2} \sum_{JK} \Bigg(\sum_{pqrs} \sum_{ML} d_{pqrs} (pq|J)(J|M)^{-1}(M|L)^{(1)}(L|K)^{-1}(K|rs) \Bigg).\end{aligned} \tag{6}$$

We can now notice that the $d_N^{(1)}$ contributions to $(pq|J)^{[1]}$ and $(M|L)^{(1)}$ are:

$$(pq|J)^{[1]} = \frac{d_N^{(1)}}{N_e} (S_{pq} d_J + d_{pq} S_J) \tag{7}$$

$$(M|L)^{(1)} = \frac{d_N^{(1)}}{N_e} (S_M d_L + d_M S_L). \tag{8}$$

Then, inserting eq. (7) into the first term of eq. (6), one gets

$$\frac{d_N^{(1)}}{N_e}\sum_{pqrs} d_{pqrs}\sum_{JK}(S_{pq}d_J + d_{pq}S_J)(J|K)^{-1}(K|rs). \tag{9}$$

Now, using the identities in eq. (1), we find

$$\frac{d_N^{(1)}}{N_e}\sum_{pqrs} d_{pqrs}(\delta_{pq}d_{rs} + d_{pq}\delta_{rs}) = 2\frac{d_N^{(1)}}{N_e}\sum_{p} W_{pp}^{\text{dipole}}, \tag{10}$$

where we have used the symmetry properties of d_{pqrs} and defined $W_{pq}^{\text{dipole}} = \sum_{rs} d_{pqrs}d_{rs}$. Inserting eq. (8) in the second term of eq. (6), one finds

$$-\frac{1}{2}\frac{d_N^{(1)}}{N_e}\sum_{pqrs} d_{pqrs}\sum_{MJ}\sum_{LK}(pq|J)(J|M)^{-1}(S_M d_L + d_M S_L)(L|K)^{-1}(K|rs). \tag{11}$$

Applying the identities in eq. (1) one finds

$$-\frac{1}{2}\frac{d_N^{(1)}}{N_e}\sum_{pqrs} d_{pqrs}(\delta_{pq}d_{rs} + d_{pq}\delta_{rs}) = -\frac{d_N^{(1)}}{N_e}\sum_{p} W_{pp}^{\text{dipole}}. \tag{12}$$

Finally, the sum of the two terms gives $\frac{d_N^{(1)}}{N_e}\sum_p W_{pp}^{\text{dipole}}$. Note that this term is equivalent to the one derived without considering the Cholesky decomposition of the dressed two-electron integrals proposed in the same text.

As for the two-electron integrals contributions to the orbital relaxation terms, these can be written as:[1]

$$\sum_{aij}\bar{\kappa}_{ai}(2g_{aijj}^{[1]} - g_{ajji}^{[1]}) = \sum_{aiK} K_{ai}^{K}(ai|K)^{[1]} + \sum_{jK} L^{K}(K|jj)^{[1]} + \sum_{jiK} N_{ji}^{K}(K|ji)^{[1]} \tag{13}$$

$$-\sum_{KL} M_{KL}(K|L)^{(1)} - \sum_{KL} O_{KL}(K|L)^{(1)} \tag{14}$$

where we have used the intermediates[1]

$$K_{ai}^{K} = \textstyle\sum_j (2\bar{\kappa}_{ai}Z_{jj}^{K} - \bar{\kappa}_{aj}Z_{ij}^{K}) \qquad N_{ji}^{K} = -\textstyle\sum_a \bar{\kappa}_{ai}Z_{aj}^{K}$$

$$L^{K} = 2\textstyle\sum_{ai}\bar{\kappa}_{ai}Z_{ai}^{K}$$

$$M_{KL} = \textstyle\sum_j L^{K}Z_{jj}^{L} \qquad O_{KL} = \textstyle\sum_{ij} N_{ji}^{K}Z_{ji}^{L}.$$

Using the definitions of the different intermediates and only considering the $d_N^{(1)}$ contributions to the derivatives, we get for the first term:

$$\frac{d_N^{(1)}}{N_e}\left(\sum_{aij} 2\bar{\kappa}_{ai}d_{ai}\sum_K S_K Z_{jj}^{K} - \sum_{aij}\bar{\kappa}_{aj}d_{ai}\sum_K S_K Z_{ij}^{K}\right) \tag{15}$$

$$=\frac{d_N^{(1)}}{N_e}\left(N_e\sum_{ai}\bar{\kappa}_{ai}d_{ai} - \sum_{ai}\bar{\kappa}_{ai}d_{ai}\right) = d_N^{(1)}\left(1 - \frac{1}{N_e}\right)\left(\sum_{ai}\bar{\kappa}_{ai}d_{ai}\right) \tag{16}$$

while for the second term:

$$2\frac{d_N^{(1)}}{N_e}\left(\sum_{aij}\bar{\kappa}_{ai}d_{jj}\sum_K S_K Z_{ai}^K+\sum_{aij}\bar{\kappa}_{ai}\delta_{jj}\sum_K d_K Z_{ai}^K\right)=d_N^{(1)}\sum_{ai}\bar{\kappa}_{ai}d_{ai}. \tag{17}$$

For the third term:

$$-\frac{d_N^{(1)}}{N_e}\left(\sum_{aij}\bar{\kappa}_{ai}d_{ji}\sum_K S_K Z_{aj}^K+\sum_{aij}\bar{\kappa}_{ai}S_{ji}\sum_K d_K Z_{aj}^K\right)=-\frac{d_N^{(1)}}{N_e}\sum_{ai}\bar{\kappa}_{ai}d_{ai}. \tag{18}$$

For the fourth term:

$$-2\frac{d_N^{(1)}}{N_e}\sum_{aij}\bar{\kappa}_{ai}\left(\sum_{KL}Z_{ai}^K S_K d_L Z_{jj}^L+\sum_{KL}Z_{ai}^K d_K S_L Z_{jj}^L\right)=-d_N^{(1)}\sum_{ai}\bar{\kappa}_{ai}d_{ai}. \tag{19}$$

Finally, for the fifth term we find:

$$\frac{d_N^{(1)}}{N_e}\sum_{aij}\bar{\kappa}_{ai}\left(\sum_{KL}Z_{aj}^K S_K d_L Z_{ji}^L+\sum_{KL}Z_{aj}^K d_K S_L Z_{ji}^L\right)=\frac{d_N^{(1)}}{N_e}\sum_{ai}\bar{\kappa}_{ai}d_{ai}. \tag{20}$$

Where we have made extensive use of the identities in eq. (1). Note that the second and the fourth terms cancels each other out, as well as the third and the fifth. As a consequence, the $d_N^{(1)}$ contributions to the orbital relaxation gradient can be expressed as

$$\sum_{aij}\bar{\kappa}_{ai}(2g_{aijj}^{[1]}-g_{ajji}^{[1]})=d_N^{(1)}\left(1-\frac{1}{N_e}\right)\left(\sum_{ai}\bar{\kappa}_{ai}d_{ai}\right) \tag{21}$$

which is equivalent (up to the chosen threshold for the Cholesky decomposition) to the expression obtained without differentiating the Cholesky decomposition.

S3. DERIVATIONS FOR THE REORTHONORMALIZATION CONTRIBUTIONS

The reorthonormalization terms in the gradient are defined as the contributions arising from the one-index transformations of the Hamiltonian and the Fock matrix. This is defined as:

$$h_{pq}^{\{1\}}=\{S^{[1]},h\}_{pq}=\sum_t S_{pt}^{[1]}h_{tq}+S_{qt}^{[1]}h_{pt}. \tag{22}$$

In the evaluation of the gradient, terms of this kind are contracted with the densities or the orbital relaxation multipliers $\bar{\kappa}_{ai}$, and we can rewrite these contributions as expressions of the form $-\sum_{pq}S_{pq}^{[1]}\mathcal{F}_{pq}$. In the following, we provide derivations of the first two terms of the generalized Fock matrix presented in the main text in eq. (59). Expressions of $\mathscr{F}_{pq}^{\bar{\kappa},2e}$ can be found in Ref. 1 and are omitted here.

A. Dressed one-electron integral

The dressed one-electron integral is defined as

$$h_{pq} = h^e_{pq} + \frac{1}{2}\sum_r d_{pr}d_{rq} - \langle d\rangle d_{pq} + \frac{\delta_{pq}}{2N_e}\langle d\rangle^2. \tag{23}$$

In the following, we will analyze all the contributions coming from the different terms of this integral. We will use the notation $\mathscr{F}_{pq}$ += to indicate contributions to add to $\mathscr{F}_{pq}$.
The reorthonormalization contributions for the purely electronic part are derived as:

$$\begin{aligned}\sum_{pq} D^e_{pq}h^{\{1\}}_{pq} = \sum_{pqt}\left(D^e_{pq}S^{[1]}_{pt}h_{tq} + D^e_{pq}S^{[1]}_{qt}h_{pt}\right) &= \sum_{pq} S^{[1]}_{pq}\left(\sum_r \mathcal{D}^e_{pr}h_{rq}\right)\\ &\Rightarrow \mathscr{F}_{pq} \mathrel{+}= \left(\sum_r \mathcal{D}^e_{pr}h_{rq}\right)\end{aligned} \tag{24}$$

where in the last equality we have redefined $p \leftrightarrow q$ and used the symmetry of h_{pq}. Moreover, we have introduced the symmetrized density matrix $\mathcal{D}^e_{pq} = D^e_{pq} + D^e_{qp}$.

For the term coming from the dipole self-energy $\frac{1}{2}\sum_r d_{pr}d_{rq}$ we have:

$$\left(\sum_r d_{pr}d_{rq}\right)^{\{1\}} = \sum_r \left(d_{pr}d^{\{1\}}_{rq} + d^{\{1\}}_{pr}d_{rq}\right) \tag{25}$$

$$\begin{aligned}\sum_{pq} D^e_{pq}\left(\sum_r d_{pr}d_{rq}\right)^{\{1\}} &= \sum_{pqr}\left(D^e_{pq}d_{pr}d^{\{1\}}_{rq} + D^e_{pq}d^{\{1\}}_{pr}d_{rq}\right) = \sum_{pqr}\mathcal{D}^e_{pq}d_{pr}d^{\{1\}}_{rq}\\ &= \sum_{pqrt}\mathcal{D}^e_{pq}d_{pr}S^{[1]}_{rt}d_{tq} + \mathcal{D}^e_{pq}d_{pr}S^{[1]}_{qt}d_{rt}\\ &= \sum_{pq}S^{[1]}_{pq}\left(\sum_{rs}\mathcal{D}^e_{rs}d_{pr}d_{sq} + \mathcal{D}^e_{pr}d_{rs}d_{sq}\right)\\ &\Rightarrow \mathscr{F}_{pq} \mathrel{+}= \sum_{rs}\mathcal{D}^e_{rs}d_{pr}d_{sq} + \sum_{rs}\mathcal{D}^e_{pr}d_{rs}d_{sq}.\end{aligned} \tag{26}$$

For the $\langle d\rangle d_{pq}$ term, instead, we get:

$$\begin{aligned}(\langle d\rangle d_{pq})^{\{1\}} &= \langle d\rangle^{\{1\}}d_{pq} + \langle d\rangle d^{\{1\}}_{pq}\\ \sum_{pq} D^e_{pq}(\langle d\rangle d_{pq})^{\{1\}} &= \sum_{pq} D^e_{pq}\langle d\rangle^{\{1\}}d_{pq} + \sum_{pq} D^e_{pq}\langle d\rangle d^{\{1\}}_{pq}.\end{aligned} \tag{27}$$

From the first term we get

$$\langle d\rangle^{\{1\}} = \sum_i 2d_{ii}^{\{1\}} = 2\sum_i \{S^{[1]}, d\}_{ii} = 2\sum_{it} S_{it}^{[1]} d_{ti} + S_{it}^{[1]} d_{it} = 4\sum_{it} S_{ti}^{[1]} d_{ti}$$

$$\sum_{pq} D_{pq}^e \langle d\rangle^{\{1\}} d_{pq} = \sum_{it} S_{ti}^{[1]} \left(4d_{ti} \sum_{pq} D_{pq}^e d_{pq} \right) \quad \Rightarrow \quad \mathscr{F}_{pi}^{\langle d\rangle} \mathrel{+}= 4d_{pi} \sum_{rs} D_{rs}^e d_{rs}. \tag{28}$$

While from the second term we get

$$\sum_{pq} D_{pq}^e \langle d\rangle d_{pq}^{\{1\}} = \sum_{pq} S_{pq}^{[1]} \left(\langle d\rangle \sum_r \mathcal{D}_{pr}^e d_{rq} \right) \quad \Rightarrow \quad \mathscr{F}_{pq} \mathrel{+}= \langle d\rangle \sum_r \mathcal{D}_{pr}^e d_{rq}. \tag{29}$$

Finally, for the $\langle d\rangle^2$ term we have

$$\frac{\delta_{pq}}{2N_e} (\langle d\rangle^2)^{\{1\}} = \frac{\delta_{pq}}{N_e} \langle d\rangle \langle d\rangle^{\{1\}} = 4\frac{\delta_{pq}}{N_e} \langle d\rangle \sum_{it} S_{ti}^{[1]} d_{ti}$$

$$\sum_{pq} D_{pq}^e \frac{\delta_{pq}}{2N_e} (\langle d\rangle^2)^{\{1\}} = \sum_p \frac{D_{pp}^e}{2N_e} (\langle d\rangle^2)^{\{1\}} = \frac{1}{2} (\langle d\rangle^2)^{\{1\}} = \langle d\rangle \langle d\rangle^{\{1\}} = 4\langle d\rangle \sum_{it} S_{ti}^{[1]} d_{ti} \tag{30}$$

$$\Rightarrow \mathscr{F}_{pi}^{\langle d\rangle} \mathrel{+}= 4\langle d\rangle d_{pi}.$$

Collecting the different terms, we can now write

$$\mathscr{F}_{pq} = \sum_r \mathcal{D}_{pr}^e h_{rq} + \frac{1}{2} \sum_{rs} \mathcal{D}_{rs}^e d_{pr} d_{sq} - \frac{\mathcal{D}_{pq}^e}{2N_e} \langle d\rangle^2$$

$$\mathscr{F}_{pi}^{\langle d\rangle} = -4d_{pi} \sum_{rs} D_{rs}^e d_{rs} + 4\langle d\rangle d_{pi} \tag{31}$$

where

$$\sum_r \mathcal{D}_{pr}^e h_{rq} = \sum_r \mathcal{D}_{pr}^e h_{rq}^e + \frac{1}{2} \sum_{rs} \mathcal{D}_{pr}^e d_{rs} d_{sq} - \langle d\rangle \sum_r \mathcal{D}_{pr}^e d_{rq} + \frac{\mathcal{D}_{pq}^e}{2N_e} \langle d\rangle^2. \tag{32}$$

B. Reorthonormalization of the bilinear term

The bilinear term in the Hamiltonian $\sqrt{\frac{\omega}{2}} \sum_{pq} d_{pq} E_{pq} (b^\dagger + b)$ leads to reorthonormalization terms that are:

$$\sqrt{\frac{\omega}{2}} \sum_{pq} D_{pq}^{e\text{-}p} d_{pq}^{\{1\}} \Rightarrow \mathscr{F}_{pq} \mathrel{+}= \sqrt{\frac{\omega}{2}} \sum_r \mathcal{D}_{pr}^{e\text{-}p} d_{rq} \tag{33}$$

and

$$-\sqrt{\frac{\omega}{2}} D^p \langle d\rangle^{\{1\}} = -4\sqrt{\frac{\omega}{2}} D^p \sum_{it} S_{ti}^{[1]} d_{ti} \Rightarrow \mathscr{F}_{pi}^{\langle d\rangle} \mathrel{+}= -4\sqrt{\frac{\omega}{2}} D^p d_{pi} \tag{34}$$

where we have reused the results for $d_{pq}^{\{1\}}$ and $\langle d\rangle^{\{1\}}$ derived in the previous section.

C. Orbital relaxation (Fock matrix)

Finally, we derive the reorthonormalization contributions coming from the derivative of the Fock matrix. As the dressed two-electron integrals are decomposed in terms of Cholesky vectors, their contributions are automatically included using the implementation proposed in Ref. 1 with a proper redefinition of the Cholesky vectors. For this reason, we here focus only on the terms arising from the one-electron integrals.

Here, we define an extended matrix $\bar{\boldsymbol{\kappa}}$ as:

$$\bar{\boldsymbol{\kappa}} = \begin{pmatrix} 0 & 0 \\ \bar{\kappa}_{ai} & 0 \end{pmatrix} \tag{35}$$

We can now derive the reorthonormalization terms as:

$$\sum_{ai} \bar{\kappa}_{ai} h_{ai}^{\{1\}} = \sum_{pq} \bar{\kappa}_{pq} h_{pq}^{\{1\}} \tag{36}$$

And, applying the same procedure we used for the one-electron integrals contributions we get:

$$\begin{aligned} \mathscr{F}_{pq} &\mathrel{+}= \sum_r \bar{\kappa}^s_{pr} h_{rq} + \frac{1}{2}\sum_{rs} \bar{\kappa}^s_{rs} d_{pr} d_{sq} - \frac{\bar{\kappa}^s_{pq}}{2N_e}\langle d\rangle^2 \\ \mathscr{F}^{\langle d\rangle}_{pi} &\mathrel{+}= -4d_{pi}\sum_{rs}\bar{\kappa}_{rs} d_{rs} + 4\sum_p \frac{\bar{\kappa}_{pp}}{N_e}\langle d\rangle d_{pi} \end{aligned} \tag{37}$$

Here, we have introduced a symmetrized $\bar{\boldsymbol{\kappa}}$ defined as $\bar{\kappa}^s_{pq} = \bar{\kappa}_{pq} + \bar{\kappa}_{qp}$, using the definition of $\bar{\boldsymbol{\kappa}}$ given in eq. (35).

We can now note that $\bar{\kappa}_{pp} = 0$ and define some extended densities $D^{\bar{\kappa}}_{pq}$ and their symmetrized analogs $\mathcal{D}^{\bar{\kappa}}_{pq}$ as

$$D^{\bar{\kappa}}_{pq} = D_{pq} + \bar{\kappa}_{pq} \qquad \mathcal{D}^{\bar{\kappa}}_{pq} = D^{\bar{\kappa}}_{pq} + D^{\bar{\kappa}}_{qp} \tag{38}$$

This allows us to derive the final expressions for $\mathscr{F}_{pq}$ and $\mathscr{F}^{\langle d\rangle}_{pq}$:

$$\begin{aligned} \mathscr{F}_{pq} &= \sum_r \left[\mathcal{D}^{\bar{\kappa}}_{pr} h_{rq} + \sum_J \tilde{\mathcal{W}}^J_{pr} L^J_{qr}\right] + \frac{1}{2}\sum_{rs}\mathcal{D}^{\bar{\kappa}}_{rs} d_{pr} d_{sq} - \frac{\langle d\rangle^2}{2N_e}\mathcal{D}^{\bar{\kappa}}_{pq} + \sqrt{\frac{\omega}{2}}\sum_r \mathcal{D}^{e\text{-}p}_{pr} d_{rq} \\ \mathscr{F}^{\langle d\rangle}_{pi} &= -4d_{pi}\left(\sum_{rs} D^{\bar{\kappa}}_{rs} d_{rs} - \langle d\rangle - \sqrt{\frac{\omega}{2}} D^p\right) \\ \mathscr{F}^{\langle d\rangle}_{pa} &= 0 \end{aligned} \tag{39}$$

S4. DENSITY MATRIX EXPRESSIONS

Here, we list programmable expressions for the QED-CCSD-1 density matrices.

A. One-electron density

Occupied-Occupied block

$$D^{e}_{ij} = 2\delta_{ij} - \sum_{abk} \bar{t}_{ajbk} t_{aibk} - \sum_{a} \bar{s}_{aj} s_{ai} - \sum_{abk} \bar{s}_{ajbk} s_{aibk} \tag{40}$$

Virtual-Occupied block

$$D^{e}_{ai} = \bar{t}_{ai} \tag{41}$$

Occupied-Virtual block

$$D^{e}_{ia} = 2\bar{\gamma} s_{ai} + \sum_{bj} \bar{s}_{bj} v_{aibj} + \sum_{bj} \bar{t}_{bj} u_{aibj} - \sum_{bjck} \bar{s}_{bjck} s_{aj} t_{bick} - \sum_{bjck} \bar{s}_{bjck} s_{bi} t_{ajck} \tag{42}$$

Virtual-Virtual block

$$D^{e}_{ab} = \sum_{i} \bar{s}_{ai} s_{bi} + \sum_{icj} \bar{s}_{aicj} s_{bicj} + \sum_{icj} \bar{t}_{aicj} t_{bicj} \tag{43}$$

B. One-electron-one-photon density

Occupied-Occupied block

$$\begin{aligned} D^{e\text{-}p}_{ij} = {} & 2\delta_{ij}\bar{\gamma} + 2\delta_{ij}\gamma + 2\sum_{ak} \delta_{ij} \bar{t}_{ak} s_{ak} + \sum_{akbl} \delta_{ij} \bar{t}_{akbl} s_{akbl} \\ & - \sum_{a} \bar{t}_{aj} s_{ai} - \sum_{a} \bar{s}_{aj} s_{ai} \gamma - \sum_{abk} \bar{s}_{ajbk} t_{aibk} - \sum_{abk} \bar{t}_{ajbk} s_{aibk} - \sum_{abk} \bar{s}_{ajbk} s_{ai} s_{bk} \\ & - \sum_{abk} \bar{s}_{ajbk} s_{aibk} \gamma - \sum_{abk} \bar{t}_{ajbk} t_{aibk} \gamma \end{aligned} \tag{44}$$

Virtual-Occupied block

$$D^{e\text{-}p}_{ai} = \bar{s}_{ai} + \bar{t}_{ai}\gamma + \sum_{bj} \bar{t}_{aibj} s_{bj} \tag{45}$$

Occupied-Virtual block

$$\begin{aligned}D_{ia}^{e\text{-}p} = {} & 2s_{ai} + 2\bar{\gamma}s_{ai}\gamma + \sum_{bj}\bar{s}_{bj}u_{aibj} + \sum_{bj}\bar{t}_{bj}v_{aibj} \\ & + 2\sum_{bj}\bar{s}_{bj}s_{ai}s_{bj} - 2\sum_{bj}\bar{s}_{bj}s_{aj}s_{bi} + \sum_{bj}\bar{s}_{bj}v_{aibj}\gamma + \sum_{bj}\bar{t}_{bj}u_{aibj}\gamma \\ & + \sum_{bjck}\bar{s}_{bjck}s_{ai}s_{bjck} - 2\sum_{bjck}\bar{s}_{bjck}s_{aj}s_{bick} - 2\sum_{bjck}\bar{s}_{bjck}s_{bi}s_{ajck} + \sum_{bjck}\bar{s}_{bjck}s_{bj}v_{aick} \\ & - \sum_{bjck}\bar{t}_{bjck}s_{aj}t_{bick} - \sum_{bjck}\bar{t}_{bjck}s_{bi}t_{ajck} + \sum_{bjck}\bar{t}_{bjck}s_{bj}u_{aick} \\ & - \sum_{bjck}\bar{s}_{bjck}s_{aj}t_{bick}\gamma - \sum_{bjck}\bar{s}_{bjck}s_{bi}t_{ajck}\gamma\end{aligned} \tag{46}$$

Virtual-Virtual block

$$\begin{aligned}D_{ab}^{e\text{-}p} = {} & \sum_{i}\bar{t}_{ai}s_{bi} + \sum_{i}\bar{s}_{ai}s_{bi}\gamma + \sum_{icj}\bar{s}_{aicj}t_{bicj} + \sum_{icj}\bar{t}_{aicj}s_{bicj} \\ & + \sum_{icj}\bar{s}_{aicj}s_{bi}s_{cj} + \sum_{icj}\bar{s}_{aicj}s_{bicj}\gamma + \sum_{icj}\bar{t}_{aicj}t_{bicj}\gamma\end{aligned} \tag{47}$$

C. One-photon density

$$D^{p} = \bar{\gamma} + \gamma + \sum_{ak}\bar{t}_{ak}s_{ak} + \frac{1}{2}\sum_{akbl}\bar{t}_{akbl}s_{akbl} \tag{48}$$

D. Two-electron density

Occupied-Occupied-Occupied-Occupied block

$$\begin{aligned}d_{ijkl}^{e} = {} & 4\delta_{ij}\delta_{kl} - 2\delta_{il}\delta_{jk} - 2\sum_{a}\delta_{kl}\bar{s}_{aj}s_{ai} + \sum_{a}\delta_{il}\bar{s}_{aj}s_{ak} + \sum_{a}\delta_{jk}\bar{s}_{al}s_{ai} - 2\sum_{a}\delta_{ij}\bar{s}_{al}s_{ak} \\ & - 2\sum_{abm}\delta_{kl}\bar{s}_{ajbm}s_{aibm} + \sum_{abm}\delta_{il}\bar{s}_{ajbm}s_{akbm} + \sum_{abm}\delta_{jk}\bar{s}_{albm}s_{aibm} - 2\sum_{abm}\delta_{ij}\bar{s}_{albm}s_{akbm} \\ & - 2\sum_{abm}\delta_{kl}\bar{t}_{ajbm}t_{aibm} + \sum_{abm}\delta_{il}\bar{t}_{ajbm}t_{akbm} + \sum_{abm}\delta_{jk}\bar{t}_{albm}t_{aibm} - 2\sum_{abm}\delta_{ij}\bar{t}_{albm}t_{akbm} \\ & + \sum_{ab}\bar{s}_{ajbl}s_{aibk} + \sum_{ab}\bar{t}_{ajbl}t_{aibk}\end{aligned} \tag{49}$$

Occupied-Occupied-Occupied-Virtual block

$$
\begin{aligned}
d^e_{ijka} = &-2\delta_{jk}\bar{\gamma}s_{ai} + 4\delta_{ij}\bar{\gamma}s_{ak} \\
&- \sum_{bl}\delta_{jk}\bar{s}_{bl}v_{aibl} + 2\sum_{bl}\delta_{ij}\bar{s}_{bl}v_{akbl} - \sum_{bl}\delta_{jk}\bar{t}_{bl}u_{aibl} + 2\sum_{bl}\delta_{ij}\bar{t}_{bl}u_{akbl} \\
&+ \sum_{blcm}\delta_{jk}\bar{s}_{blcm}s_{al}t_{bicm} - 2\sum_{blcm}\delta_{ij}\bar{s}_{blcm}s_{al}t_{bkcm} \\
&+ \sum_{blcm}\delta_{jk}\bar{s}_{blcm}s_{bi}t_{alcm} - 2\sum_{blcm}\delta_{ij}\bar{s}_{blcm}s_{bk}t_{alcm} \\
&- \sum_{b}\bar{s}_{bj}v_{akbi} - \sum_{b}\bar{t}_{bj}u_{akbi} \\
&+ \sum_{bcl}\bar{s}_{bjcl}s_{ai}t_{bkcl} - 2\sum_{bcl}\bar{s}_{bjcl}s_{ak}t_{bicl} + \sum_{bcl}\bar{s}_{bjcl}s_{al}t_{bick} \\
&+ \sum_{bcl}\bar{s}_{bjcl}s_{bk}t_{aicl} + \sum_{bcl}\bar{s}_{bjcl}s_{ck}t_{albi} - \sum_{bcl}\bar{s}_{bjcl}s_{bi}u_{akcl}
\end{aligned}
\tag{50}
$$

Occupied-Occupied-Virtual-Occupied block

$$
d^e_{ijak} = -\delta_{ik}\bar{t}_{aj} + 2\delta_{ij}\bar{t}_{ak} - \sum_{b}\bar{s}_{akbj}s_{bi} \tag{51}
$$

Occupied-Occupied-Virtual-Virtual block

$$
\begin{aligned}
d^e_{ijab} = &\,2\sum_{k}\delta_{ij}\bar{s}_{ak}s_{bk} + 2\sum_{kcl}\delta_{ij}\bar{s}_{akcl}s_{bkcl} + 2\sum_{kcl}\delta_{ij}\bar{t}_{akcl}t_{bkcl} \\
&- \bar{s}_{aj}s_{bi} - \sum_{ck}\bar{s}_{ajck}s_{bick} - \sum_{kc}\bar{s}_{akcj}s_{bkci} - \sum_{ck}\bar{t}_{ajck}t_{bick} - \sum_{kc}\bar{t}_{akcj}t_{bkci}
\end{aligned}
\tag{52}
$$

Occupied-Virtual-Occupied-Virtual block

$$
\begin{aligned}
d^{e}_{iajb} = {} & 2u_{aibj} + 2\bar{\gamma}v_{aibj} \\
& + 2\sum_{ck}\bar{s}_{ck}s_{ai}u_{bjck} - \sum_{ck}\bar{s}_{ck}s_{aj}u_{bick} - \sum_{ck}\bar{s}_{ck}s_{ak}u_{bjci} - \sum_{ck}\bar{s}_{ck}s_{bi}u_{ajck} \\
& + 2\sum_{ck}\bar{s}_{ck}s_{bj}u_{aick} - \sum_{ck}\bar{s}_{ck}s_{bk}u_{aicj} - \sum_{ck}\bar{s}_{ck}s_{ci}u_{akbj} - \sum_{ck}\bar{s}_{ck}s_{cj}u_{aibk} \\
& + \sum_{ckdl}\bar{s}_{ckdl}s_{akbl}t_{cidj} + \sum_{ckdl}\bar{s}_{ckdl}s_{akcj}t_{bidl} + \sum_{ckdl}\bar{s}_{ckdl}s_{akdj}t_{blci} + \sum_{ckdl}\bar{s}_{ckdl}s_{bkci}t_{ajdl} \\
& + \sum_{ckdl}\bar{s}_{ckdl}s_{bkdi}t_{alcj} + \sum_{ckdl}\bar{s}_{ckdl}s_{cidj}t_{akbl} - \sum_{ckdl}\bar{s}_{ckdl}s_{ajck}u_{bidl} - \sum_{ckdl}\bar{s}_{ckdl}s_{akdl}u_{bjci} \\
& - \sum_{ckdl}\bar{s}_{ckdl}s_{bick}u_{ajdl} - \sum_{ckdl}\bar{s}_{ckdl}s_{bkdl}u_{aicj} - \sum_{ckdl}\bar{s}_{ckdl}s_{cidl}u_{akbj} - \sum_{ckdl}\bar{s}_{ckdl}s_{cjdl}u_{aibk} \\
& - \sum_{ckdl}\bar{s}_{ckdl}t_{akdl}v_{bjci} - \sum_{ckdl}\bar{s}_{ckdl}t_{bkdl}v_{aicj} - \sum_{ckdl}\bar{s}_{ckdl}t_{cidl}v_{akbj} - \sum_{ckdl}\bar{s}_{ckdl}t_{cjdl}v_{aibk} \\
& + \sum_{ckdl}\bar{s}_{ckdl}u_{aick}v_{bjdl} + \sum_{ckdl}\bar{s}_{ckdl}u_{bjck}v_{aidl} + \sum_{ckdl}\bar{t}_{ckdl}t_{ajck}t_{bldi} + \sum_{ckdl}\bar{t}_{ckdl}t_{akbl}t_{cidj} \\
& + \sum_{ckdl}\bar{t}_{ckdl}t_{akdj}t_{blci} - \sum_{ckdl}\bar{t}_{ckdl}t_{akdl}u_{bjci} - \sum_{ckdl}\bar{t}_{ckdl}t_{bick}u_{ajdl} - \sum_{ckdl}\bar{t}_{ckdl}t_{bkdl}u_{aicj} \\
& - \sum_{ckdl}\bar{t}_{ckdl}t_{cidl}u_{akbj} - \sum_{ckdl}\bar{t}_{ckdl}t_{cjdl}u_{aibk} + \sum_{ckdl}\bar{t}_{ckdl}u_{aick}u_{bjdl}
\end{aligned}
\tag{53}
$$

Occupied-Virtual-Virtual-Occupied block

$$
\begin{aligned}
d^{e}_{iabj} = {} & -\sum_{k}\delta_{ij}\bar{s}_{bk}s_{ak} - \sum_{kcl}\delta_{ij}\bar{s}_{bkcl}s_{akcl} - \sum_{kcl}\delta_{ij}\bar{t}_{bkcl}t_{akcl} \\
& + 2\bar{s}_{bj}s_{ai} + \sum_{ck}\bar{s}_{bjck}v_{aick} + \sum_{ck}\bar{t}_{bjck}u_{aick}
\end{aligned}
\tag{54}
$$

Occupied-Virtual-Virtual-Virtual block

$$
\begin{aligned}
d^{e}_{iabc} = {} & \sum_{j}\bar{s}_{bj}v_{aicj} + \sum_{j}\bar{t}_{bj}u_{aicj} \\
& + 2\sum_{jdk}\bar{s}_{bjdk}s_{ai}t_{cjdk} - \sum_{jdk}\bar{s}_{bjdk}s_{aj}t_{cidk} - \sum_{jdk}\bar{s}_{bjdk}s_{ak}t_{cjdi} \\
& - \sum_{jdk}\bar{s}_{bjdk}s_{ci}t_{ajdk} - \sum_{jdk}\bar{s}_{bjdk}s_{di}t_{akcj} + \sum_{jdk}\bar{s}_{bjdk}s_{cj}u_{aidk}
\end{aligned}
\tag{55}
$$

Virtual-Occupied-Virtual-Occupied block

$$
d^{e}_{aibj} = \bar{t}_{aibj} \tag{56}
$$

Virtual-Occupied-Virtual-Virtual block

$$d^e_{aibc} = \sum_j \bar{s}_{aibj} s_{cj} \tag{57}$$

Virtual-Virtual-Virtual-Virtual block

$$d^e_{abcd} = \sum_{ij} \bar{s}_{aicj} s_{bidj} + \sum_{ij} \bar{t}_{aicj} t_{bidj} \tag{58}$$

REFERENCES

[1] A. K. Schnack-Petersen, H. Koch, S. Coriani, and E. F. Kjønstad, "Efficient implementation of molecular CCSD gradients with Cholesky-decomposed electron repulsion integrals," The Journal of Chemical Physics **156**, 244111 (2022), https://pubs.aip.org/aip/jcp/article-pdf/doi/10.1063/5.0087261/16545294/244111_1_online.pdf.
